# Acknowledgement of manuscript reviewers 2014

**DOI:** 10.1186/s12966-015-0170-z

**Published:** 2015-03-12

**Authors:** Russell Jago, Frank van Lenthe

**Affiliations:** University of Bristol, ᅟ, United Kingdom

## Abstract

The editors of International Journal of Behavioral Nutrition and Physical Activity would like to thank all our reviewers who have contributed to the journal in Volume 11 (2014).

**Suzanne Abraham**

Australia

**Anna Adachi-Mejia**

United States of America

**Emma Adams**

United Kingdom

**Minna Aittasalo**

Finland

**Annie Anderson**

United Kingdom

**Andrew J Atkin**

United Kingdom

**Suzanne Audrey**

United Kingdom

**Garry Auld**

United States of America

**Mauricio Avendano**

United States of America

**Helena Baert**

United States of America

**Graham Baker**

United Kingdom

**Katherine Baldock**

Australia

**Austin Baldwin**

United States of America

**Kylie Ball**

Australia

**Tom Baranowski**

United States of America

**Sally Barber**

United Kingdom

**Cristina Barroso**

United States of America

**Jimikaye Beck**

United States of America

**Rebecca Beeken**

United Kingdom

**Marielle Beenackers**

Netherlands

**Christie Befort**

United States of America

**Elling Bere**

Norway

**Jerica Berge**

United States of America

**Claire Bernaards**

Netherlands

**David Berrigan**

United States of America

**Tanya Berry**

Canada

**Kathelijne Bessems**

Netherlands

**Hervé Besson**

United Kingdom

**Prachi Bhatnagar**

United Kingdom

**Stuart Biddle**

United Kingdom

**Amanda Birnbaum**

United States of America

**Maureen Black**

United States of America

**Christine Blake**

United States of America

**Filip Boen**

Belgium

**Lars Borghouts**

Netherlands

**Daniel Bornstein**

United States of America

**Judit Bort-Roig**

Spain

**Adam Briggs**

United Kingdom

**Anna Broberg**

Finland

**Justin Brown**

United States of America

**Helen Elizabeth Brown**

United Kingdom

**Stephanie Broyles**

United States of America

**Johannes Brug**

Netherlands

**Nicola Buckland**

United Kingdom

**Jens Bucksch**

Germany

**Nicola Burton**

Australia

**Lorraine Cale**

United Kingdom

**Robin Callister**

Australia

**Adrian Cameron**

Australia

**Susan Carnell**

United Kingdom

**Megan Ann Carter**

Canada

**Alison Carver**

Australia

**Gianluca Castelnuovo**

Italy

**Carlos Celis Morales**

United Kingdom

**Catherine Chan**

Canada

**Sebastien Chastin**

United Kingdom

**Mai Jeanette Maidy Chinapaw**

Netherlands

**Bronwyn Clark**

Australia

**Verity Cleland**

Australia

**Natalie Colabianchi**

United States Minor Outlying Islands

**Lucy Cooke**

United Kingdom

**Dean Cooley**

Australia

**Emma Coombes**

United Kingdom

**Angela Craigie**

United Kingdom

**David Crawford**

Australia

**Noe Crespo**

United States of America

**Matthew Cross**

United States of America

**Rik Crutzen**

Netherlands

**Jayna Dave**

United States of America

**R Sue Day**

United States of America

**Joop De Boer**

Netherlands

**Ilse De Bourdeaudhuij**

Belgium

**Emely De Vet**

Netherlands

**Petra De Vries**

Netherlands

**Benedicte Deforche**

Belgium

**Ingrid Demmelmaier**

Sweden

**Katharine Wickel Didericksen**

United States of America

**Rod Dishman**

United States of America

**Beth Dixon**

United States of America

**Aiden Doherty**

United Kingdom

**James Dollman**

Australia

**Catherine Draper**

South Africa

**Kimberly Drews**

United States of America

**Shufa Du**

United States of America

**Mitch J Duncan**

Australia

**Scott Duncan**

New Zealand

**Kerith Duncanson**

Australia

**David Dzewaltowski**

United States of America

**Elizabeth Eakin**

Australia

**Narelle Eather**

Australia

**Lynn Edmunds**

United States of America

**Rochelle Eime**

Australia

**Liselotte Schäfer Elinder**

Sweden

**Pauline Emmett**

United Kingdom

**Clare England**

United Kingdom

**Michelle Estrade**

United Kingdom

**Astrid Etman**

Netherlands

**Kelly Evenson**

United States of America

**Nicole Ezendam**

Netherlands

**Stuart Fairclough**

United Kingdom

**Jennifer Falbe**

United States of America

**Barbara Fiese**

United States of America

**Abi Fisher**

United Kingdom

**Natasha Franklin**

Australia

**Laurence Freedman**

Israel

**Marjorie Freedman**

United States of America

**Stijn Friederichs**

Netherlands

**Paul Fuglestad**

United States of America

**Jayne Fulkerson**

United States of America

**Bridget Gaglio**

United States of America

**Amy Galloway**

United States of America

**Kim Gans**

United States of America

**Enrique Garcia Bengoechea**

Canada

**Paul Gardiner**

Australia

**Benjamin Gardner**

United Kingdom

**Emma Gearon**

Australia

**Klaus Gebel**

Australia

**Simone Gill**

United States of America

**Meghan Gillen**

United States of America

**Fiona Gillison**

United Kingdom

**Karen Glanz**

United States of America

**Rebecca Golley**

Australia

**Lora Suzanne Goodell**

United States of America

**Gerlinde Grasser**

Austria

**Cindy Gray**

United Kingdom

**Alison Gustafson**

United States of America

**Josephine Gwynn**

Australia

**Anne Haase**

United Kingdom

**Jess Haines**

Canada

**Pedro Hallal**

Brazil

**Mark Hamer**

United Kingdom

**Katherine Hanna**

Australia

**Louise Hardy**

Australia

**Deirdre Harrington**

United Kingdom

**Richard Harrington**

United Kingdom

**Flo Harrison**

United Kingdom

**Peter Hart**

United States of America

**Sheri Hartman**

United States of America

**Marieke Antonia Hartman**

United States of America

**Emma Haycraft**

United Kingdom

**Sharon Hayes**

United States of America

**Genevieve Healy**

Australia

**Gregory Heath**

United States of America

**Eva Heinen**

United Kingdom

**Ilkka Heinonen**

Netherlands

**Erik Hemmingsson**

Sweden

**Karla Henderson**

United States of America

**Erin Hennessy**

United States of America

**Kylie Hesketh**

Australia

**David Hey**

United States of America

**Melvyn Hillsdon**

United Kingdom

**Erica Hinckson**

New Zealand

**Melanie Hingle**

United States of America

**Mads Hjorth**

Denmark

**Mandy Ho**

Hong Kong

**Eric Hodges**

United States of America

**Bjørn E Holstein**

Denmark

**Andreas Holtermann**

Denmark

**Ciska Hoving**

Netherlands

**Wendy Yajun Huang**

Hong Kong

**M Louise Humbert**

Canada

**Jean Hunleth**

United States of America

**Anita Hurtig-Wennlof**

Sweden

**Ronald Iannotti**

United States of America

**April Idalski Carcone**

United States of America

**Shigeru Inoue**

Japan

**Brandon Irwin**

United States of America

**Janet James**

United Kingdom

**Elena Jansen**

Australia

**Maria Jansen**

Netherlands

**Robert W Jeffery**

United States of America

**Megan Jensen**

Canada

**Laura Johnson**

United Kingdom

**Rebecca Johnson**

United States of America

**Susan Johnson**

United States of America

**Kate Jolly**

United Kingdom

**Andy Jones**

United Kingdom

**Andrew T. Kaczynski**

United States of America

**Sonja Kahlmeier**

Switzerland

**Carlijn Kamphuis**

Netherlands

**Sharon Karp**

United States of America

**Paul Kelly**

United Kingdom

**Jacqueline Kerr**

United States of America

**Jean Kerver**

United States of America

**Joanna Kesten**

United Kingdom

**Ruth Kipping**

United Kingdom

**Joanna Kirby**

United Kingdom

**Heather Kitzman-Ulrich**

United States of America

**Knut-Inge Klepp**

Norway

**Nicolas Knuth**

United States of America

**Gerjo Kok**

Netherlands

**Tracy Kolbe-Alexander**

Australia

**Sibylle Kranz**

United Kingdom

**Katja Kröller**

Germany

**Martha Kubik**

United States of America

**Anton Kunst**

Netherlands

**Lydia Kwak**

Sweden

**Jennifer La Guardia**

United States of America

**Kathleen Lacy**

Australia

**Jouni Lahti**

Finland

**Jeroen Lakerveld**

Netherlands

**Karen Lamb**

Australia

**Amy Lampard**

United States of America

**Anthony Laverty**

United Kingdom

**Rachel Laws**

Australia

**Ha Le**

Australia

**Emma Lea**

Australia

**Amanda Lee**

Australia

**Heather Leidy**

United States of America

**Stephenie Lemon**

United States of America

**Alastair Leyland**

United Kingdom

**Kaigang Li**

United States of America

**Nanna Lien**

Norway

**Barbara Lohse**

United States of America

**Chris Lonsdale**

Australia

**Theo Lorenc**

United Kingdom

**David Lubans**

Australia

**Sean Lucan**

United States of America

**Anne Lusk**

United States of America

**Elizabeth Lyons**

United States of America

**Ruth Mabry**

Oman

**Kyle Macdonald-Wallis**

United Kingdom

**Graham Macgregor**

United Kingdom

**Joreintje Mackenbach**

Netherlands

**Roger Mackett**

United Kingdom

**Freya Macmillan**

Australia

**Lea Maes**

Belgium

**Anthea Magarey**

Australia

**Emily Mailey**

United States of America

**Jennifer Marks**

Australia

**Marta Marques**

Portugal

**Louise C Masse**

Canada

**Sarah Mcnaughton**

Australia

**Amy Mcpherson**

Canada

**Melissa Melby**

United States of America

**Robin Mellecker**

Hong Kong

**Jason Mendoza**

United States of America

**Dafna Merom**

Australia

**Brad Metcalf**

United Kingdom

**Anouk Middelweerd**

Netherlands

**Bent Egberg Mikkelsen**

Denmark

**Josef Mitas**

Czech Republic

**Richard Mitchell**

United Kingdom

**Sandrine Monnery-Patris**

France

**Jesse Morrell**

United States of America

**Niamh Murphy**

Ireland

**Patti-Jean Naylor**

Canada

**Theresa Nicklas**

United States of America

**Glen Nielsen**

Denmark

**Faryle Nothwehr**

United States of America

**Paulina Nowicka**

Sweden

**Caryl Nowson**

Australia

**Gisela Nyberg**

Sweden

**Julie Obbagy**

United States of America

**Teresia O'Connor**

United States of America

**Angela Odoms-Young**

United States of America

**David Ogilvie**

United Kingdom

**Tim Olds**

Australia

**Melody Oliver**

New Zealand

**Christine Olson**

United States of America

**Jennifer O'Neill**

United States of America

**Jean-Michel Oppert**

France

**Nicolas Oreskovic**

United States of America

**Juliane Paech**

Germany

**Jenna Panter**

United Kingdom

**Angeliki Papadaki**

United Kingdom

**Sanjoti Parekh**

Australia

**Diana Parra**

Brazil

**Russell Pate**

United States of America

**Amanda Patterson**

Australia

**Jamie Pearce**

United Kingdom

**Rebecca Pearl**

United States of America

**Anna Peeters**

Australia

**Louisa Peralta**

Australia

**Karin Pfeiffer**

United States of America

**Lisa Phillips**

United Kingdom

**Claudia R Pischke**

Germany

**Astrid Poelman**

Australia

**Gina Porter**

United Kingdom

**Thomas Power**

United States of America

**Richard G. Prins**

Netherlands

**Karin Proper**

Netherlands

**Pekka Puska**

Finland

**Merja Rantakokko**

Finland

**Gail Regan**

United States of America

**Dominique Reinwand**

Germany

**Teun Remmers**

Netherlands

**Kristina Martha Renault**

Denmark

**Kyung Rhee**

United States of America

**Ryan Rhodes**

Canada

**Justin Richards**

Australia

**Tracy Richmond**

United States of America

**Nicola Diane Ridgers**

Australia

**Chris Rissel**

Australia

**Lorrene Ritchie**

United States of America

**Christina Roberto**

United States of America

**Jennifer Robertson-Wilson**

Canada

**Leah Robinson**

United States of America

**Erin Roche**

United States of America

**Carol Rodgers**

Canada

**Megan Rollo**

Australia

**Anne Rongen**

Netherlands

**Eva Roos**

Finland

**Dori Rosenberg**

United States of America

**Richard Rosenkranz**

United States of America

**Ash Routen**

United Kingdom

**Alex Rowlands**

United Kingdom

**RAC Ruiter**

Netherlands

**Zoe Rutherford**

United Kingdom

**Geert Rutten**

Netherlands

**Gemma Ryde**

United Kingdom

**Arja Sääkslahti**

Finland

**Jo Salmon**

Australia

**Ferdinand Salonna**

Czech Republic

**Deborah Salvo**

Mexico

**Julie Saunders**

Australia

**Ruth Saunders**

United States of America

**Hans Savelberg**

Netherlands

**Jasper Schipperijn**

Denmark

**Stephanie Schoeppe**

Australia

**Daniela Schulz**

Netherlands

**John Schuna**

United States of America

**Claire Schwartz**

United Kingdom

**Marlene Schwartz**

United States of America

**Simon Sebire**

United Kingdom

**Jan Seghers**

Belgium

**Rebecca Seguin**

United States of America

**Elena Serrano**

United States of America

**Shannon Siegel**

United States of America

**Erik Sigmund**

Czech Republic

**Amy Silva-Smith**

United States of America

**Susan Slade**

Australia

**Sandy Slater**

United States of America

**Lee Smith**

United Kingdom

**Falko F Sniehotta**

United Kingdom

**Marita Sodergren**

Australia

**Kimmo Sorjonen**

Sweden

**Alison Spence**

Australia

**Andrew Springer**

United States of America

**Tonje Holte Stea**

Norway

**Jostein Steene-Johannessen**

Norway

**Amy Storfer-Isser**

United States of America

**Vera Storm**

Germany

**Kristi Storti**

United States of America

**Gareth Stratton**

United Kingdom

**Jorien Strijk**

Netherlands

**Takemi Sugiyama**

Australia

**Carolyn Summerbell**

United Kingdom

**Chalida Svastisalee**

Denmark

**Daniel Taber**

United States of America

**Marian Tanofsky-Kraff**

United States of America

**Sharon Taverno Ross**

United States of America

**Natalie Taylor**

Australia

**David Taylor-Robinson**

United Kingdom

**Saskia Te Velde**

Netherlands

**Pedro Teixeira**

Portugal

**Richard Telford**

Australia

**Debbe Thompson**

United States of America

**Janice Thompson**

United Kingdom

**Lukar Thornton**

Australia

**Idia Thurston**

United States of America

**Anna Timperio**

Australia

**Sandar Tin Tin**

New Zealand

**Sylvia Titze**

Austria

**Nick Townsend**

United Kingdom

**Mark S Tremblay**

Canada

**Margarita Triguero-Mas**

Spain

**Philip Troped**

United States of America

**Catrine Tudor-Locke**

United States of America

**Gavin Turrell**

Australia

**Lisa Tussing-Humphreys**

United States of America

**Dale Ulrich**

United States of America

**Claudia Vadeboncoeur**

United Kingdom

**Patricia Van Assema**

Netherlands

**Jelle Van Cauwenberg**

Belgium

**Vivian Van De Gaar**

Netherlands

**Hidde P Van Der Ploeg**

Netherlands

**Lenneke Van Genugten**

Netherlands

**Dave Van Kann**

Netherlands

**Frank Van Lenthe**

Netherlands

**Femke Van Nassau**

Netherlands

**Mireille Van Poppel**

Netherlands

**Esther Van Sluijs**

United Kingdom

**Corneel Vandelanotte**

Australia

**Jenny Veitch**

Australia

**Alison Venn**

Australia

**Maite Verloigne**

Belgium

**Karen Villanueva**

Australia

**Lina Wahlgren**

Sweden

**Dianne Ward**

United States of America

**Jane Wardle**

United Kingdom

**Kathleen Watson**

United States of America

**Nancy Wells**

United States of America

**Li Ming Wen**

Australia

**Benedict Wheeler**

United Kingdom

**James White**

United Kingdom

**Ross Whitehead**

United Kingdom

**Eric Wickel**

United States of America

**Katrien Wijndaele**

United Kingdom

**Julianne Williams**

United Kingdom

**Lauren Williams**

Australia

**Elisabeth Winkler**

Australia

**Janet Withall**

United Kingdom

**Bente Wold**

Norway

**Luke Wolfenden**

Australia

**Lesley Wood**

United Kingdom

**Rebecca Woodruff**

United States of America

**Sarah Woodruff**

Canada

**Anthony Worsley**

Australia

**Neil Wrigley**

United Kingdom

**Sonja Yokum**

United States of America

**Shannon Zenk**

United States of America

